# Perceptual insensitivity to the modulation of interoceptive signals in depression, anxiety, and substance use disorders

**DOI:** 10.1038/s41598-021-81307-3

**Published:** 2021-01-22

**Authors:** Ryan Smith, Justin S. Feinstein, Rayus Kuplicki, Katherine L. Forthman, Jennifer L. Stewart, Martin P. Paulus, Tulsa 1000 Investigators, Robin L. Aupperle, Jerzy Bodurka, Jonathan B. Savitz, Teresa A. Victor, Sahib S. Khalsa

**Affiliations:** 1 Laureate Institute for Brain Research, 6655 S Yale Ave, Tulsa, OK 74136 USA; 2 Oxley College of Health Sciences, University of Tulsa, Tulsa, OK USA

**Keywords:** Psychiatric disorders, Psychology

## Abstract

This study employed a series of heartbeat perception tasks to assess the hypothesis that cardiac interoceptive processing in individuals with depression/anxiety (N = 221), and substance use disorders (N = 136) is less flexible than that of healthy individuals (N = 53) in the context of physiological perturbation. Cardiac interoception was assessed via heartbeat tapping when: (1) guessing was allowed; (2) guessing was not allowed; and (3) experiencing an interoceptive perturbation (inspiratory breath hold) expected to amplify cardiac sensation. Healthy participants showed performance improvements across the three conditions, whereas those with depression/anxiety and/or substance use disorder showed minimal improvement. Machine learning analyses suggested that individual differences in these improvements were negatively related to anxiety sensitivity, but explained relatively little variance in performance. These results reveal a perceptual insensitivity to the modulation of interoceptive signals that was evident across several common psychiatric disorders, suggesting that interoceptive deficits in the realm of psychopathology manifest most prominently during states of homeostatic perturbation.

## Introduction

There is a growing consensus that interoceptive dysfunction, and specifically dysfunction in the brain’s ability to adaptively perceive and regulate the internal state of the body, may play an important role across a range of psychiatric disorders^1^. Diagnostic criteria for mental disorders are in many cases related to interoceptive and visceral symptoms, and several components within current psychotherapeutic treatments include interoceptive interventions^2–6^. A growing number of neuroimaging meta-analyses have also implicated the insula, anterior cingulate, and amygdala as regions with altered processing in several psychiatric disorders^7–9^, and each of these regions is known to play an important role in the perception and/or regulation of internal bodily states^10,11^. Yet, the precise mechanisms underlying interoceptive dysfunction within mental disorders remain insufficiently characterized.

One widely studied interoceptive domain is cardiac perception (cardioception). A number of tasks have been developed to study this function. Although they involve the performance of different actions, including heartbeat counting, heartbeat tapping, and heartbeat detection^12–17^, the active components of these tasks all involve the shifting of goal-directed attention towards the heartbeat signal, and providing an explicit report of the perceptual experience of heartbeat sensations. Each of these tasks has a number of strengths and limitations^18–21^. For example, scores on the heartbeat counting task have been suggested to be biased by prior knowledge of heart rate^22^ and to correlate with measured heart rate^21^ (but see Ref.^23^ for a response, and Ref.^24^ for a counter-response). Regardless of one’s position on the optimal measure of cardioception, a consistent finding is that cardiac interoceptive accuracy is quite limited in the majority of individuals during resting conditions—where only roughly 35% of individuals appear to accurately perceive their own heartbeats^25^. This is true even in the case of orthoptic heart transplantation, when the heart being perceived is not the one the person was born with^26^. In contrast, heartbeat perception has been shown to increase in accuracy under conditions of heightened physiological arousal—suggesting that physiological context can play a moderating role^27^.

A related picture is beginning to emerge within clinical populations as well. While some neuroimaging studies have suggested altered interoceptive processing in psychiatric populations during interoceptive attention^28–34^, available behavioral studies of individual differences in interoceptive awareness within clinical populations have thus far been inconclusive. For example, studies of panic disorder patients under resting conditions have not demonstrated clear evidence for greater interoceptive accuracy for heartbeat sensations^35–37^, despite the fact that multiple studies have shown that panic disorder patients perceive interoceptive (cardiorespiratory) sensations more intensely during a range of different physiological perturbations that transiently amplify afferent signal strength^38–44^. These results suggest that differences in interoceptive experience during non-resting physiological contexts may be especially relevant for understanding psychopathology, especially for disorders with a heightened expression of anxiety.

In the present study, we chose to examine whether a non-invasive interoceptive perturbation would improve cardiac perception to a greater degree in healthy individuals than in a transdiagnostic sample of individuals with psychiatric disorders. We selected a transdiagnostic sample to help address the question of whether interoceptive dysfunction is disorder-specific, or common across disorders as a potential risk and/or maintenance factor. The possibility that interoceptive dysfunction is transdiagnostic is supported by current work in multiple patient populations. For example, aside from the panic disorder studies already discussed, a recent review concluded that—while work to date has a number of important limitations—the cumulative evidence suggests that individuals with moderate depression symptoms appear to show impaired interoceptive accuracy^45^. Other preliminary work has also linked alcohol use disorder with low interoceptive accuracy^46^, and there are several neuroimaging studies suggesting altered interoceptive processing within substance users^33,47–49^. Thus, we sought to confirm whether our hypothesized interoceptive processing deficit was present across these diagnostic categories.

Given the numerous criticisms of the heartbeat counting measure, the prolonged measurement duration required by heartbeat detection tasks^12,14,15,17^, and the inability to easily deploy an invasive physiological perturbation across a large sample, we employed a heartbeat tapping task^16,50^ to measure individual differences in the ability to consciously detect and behaviorally report perception of one’s own heartbeat on a beat-by-beat basis. Importantly, we adapted the deployment of this task to include both resting and physiological perturbation conditions. We expected that a non-invasive physiological perturbation of afferent cardiac signals would improve interoceptive accuracy in healthy participants and that this effect would be attenuated in psychiatric populations, perhaps reflecting a reduced capacity to modulate interoceptive processing in a flexible manner. Here we specifically chose to examine a transdiagnostic sample of individuals with mood/anxiety disorders and with substance use disorders.

## Methods

### Participants

Participants were sampled from the first 500 individuals collected in the Tulsa 1000 project^51^. The Tulsa 1000 is a naturalistic longitudinal study that recruited individuals with psychiatric disorders spanning mood, anxiety, substance use, and eating disorders with a transdiagnostic focus. Recruitment was based on the dimensional National Institute of Mental Health Research Domain Criteria framework^52^. Individuals aged 18–55 years were screened on the basis of self-reported dimensional psychopathology scores. Inclusion was based on the following measures: Patient Health Questionnaire (PHQ-9; Ref.^53^) ≥ 10, Overall Anxiety Severity and Impairment Scale (OASIS; Ref.^54^) ≥ 8, Drug Abuse Screening Test (DAST-10; Ref.^55^) score > 2, and/or Eating Disorder Screen (SCOFF; Ref.^56^) score ≥ 2. Healthy comparison participants who did not show elevated symptoms or psychiatric diagnoses were recruited during the same time period (from January 5, 2015 to February 22, 2017). For more details, see Ref.^51^. The study was approved by the Western Institutional Review Board. All participants provided written informed consent prior to completion of the study protocol, in accordance with the Declaration of Helsinki, and were compensated for participation. ClinicalTrials.gov identifier: #NCT02450240.

Due to our transdiagnostic focus on symptoms of depression, anxiety, and substance use, a subset of 410 individuals were included from the first 500 participant cohort of the Tulsa 1000. As described below, we initially examined differences between healthy participants and all patients transdiagnostically. For secondary analysis of sub-groups, patients were divided based on primary diagnoses of (1) major depressive disorder and/or co-morbid anxiety disorders, or (2) substance use disorders. Anxiety disorders included social anxiety, generalized anxiety, panic, and/or posttraumatic stress disorder. Individuals were not included in the substance use disorders group if showing only alcohol or nicotine dependence, or for whom substance use was not the primary diagnosis as determined by the clinical assessment. For previous studies using this grouping approach, see Refs.^57–59^. Participants were diagnostically grouped based on DSM-IV or DSM-5 criteria obtained from a structured clinical interview at the time of testing (Mini International Neuropsychiatric Inventory 6 or 7 (MINI; Ref.^60^). Final groups consisted of 53 healthy individuals (28 male; mean age = 31.99 ± 10.97 years), 136 individuals with a primary diagnosis of substance use disorder (60 male; mean age = 33.70 ± 8.87 years), and 221 individuals with primary diagnoses of depression and/or co-morbid anxiety disorders (62 male; mean age = 36.23 ± 11.17 years). Other participants were excluded for the following reasons: 28 had a primary diagnosis of an eating disorder (which was not the focus of the present study), 16 had problems with electrocardiogram (ECG) data collection, 19 did not follow instructions and took their pulse during at least one heartbeat perception trial (verified by examining videos of the participants), 14 had non-estimable slopes reflecting changes in task performance across task conditions (see further details about the task and associated analyses below), and 13 made fewer than 2 responses in the first task condition, which also precluded inclusion in our primary analyses (see task details below). No participants in this sample (i) tested positive for drugs of abuse, (ii) met criteria for psychotic disorders, or reported (iii) history of moderate-to-severe traumatic brain injury, neurological disorders, or severe or unstable medical conditions, (iv) active suicidal intent or plan, or (v) change in medication within 6 weeks. Consistent use of prescribed psychotropic medication was present in 68% of individuals with a primary diagnosis of depression/anxiety and in 44% of individuals with a primary diagnosis of a substance use disorder (primarily antidepressants and/or anxiolytics). For a more detailed characterization of secondary diagnoses and medications, see Supplementary Information.

### Procedure

As part of the Tulsa 1000 project, participants completed a large number of assessments, self-report measures, and behavioral tasks (detailed in Ref.^51^). Here we focus on reporting performance during one of the behavioral tasks—a “heartbeat tapping task” in which participants were asked to behaviorally indicate each moment when they perceived their heartbeat by tapping a recording device with their finger. As mentioned earlier, this design was based on similar approaches that have been previously reported^16,50^, and was selected based on a number of experimental tradeoffs. These included the need to assess cardiac interoception: (1) in a large sample of psychiatric patients with different diagnoses; (2) under a time constraint (data collection needed to be completed within a brief time period due to the large number of other tasks involved in study participation); (3) under non-invasive contexts; and (4) while still including an interoceptive perturbation likely to induce a physiological shift from a homeostatic baseline state. In the first task condition, participants were instructed to close their eyes and press a keyboard button (or touchpad in a few participants; see below) in synchrony with their heartbeat, to try to mirror their heartbeat as closely as possible. Even if they were not sure about their responses, they were told to take their best guess (the “guessing” condition), reflecting a standard instruction given during heartbeat counting tasks^61^. Participants completed this task condition over a period of 60 s. In the second task condition, all instructions were identical except that participants were told to only press the key when they actually felt their heartbeat, and if they did not feel their heartbeat then they should not press the button (the “no guessing” condition). In other words, unlike the first time they completed the task, participants were specifically instructed not to guess if they didn't confidently feel anything. They were also informed that it can be difficult to feel your heartbeat, and that some people may even go the whole trial without pressing the button at all. This “no guessing” condition can be understood as placing additional demands on the participant to monitor their own confidence in whether a heartbeat was actually felt, and, although it was not known at the time of task design, such instructions have been reported to substantially influence performance on the heartbeat counting task^62^. Participants completed this task condition over a period of 60 s. In the third (and final) task condition, participants were again instructed not to guess, and additionally were asked to first empty their lungs of all air, take as deep a breath as possible, and hold it for as long as they could tolerate (up to the length of the one-minute trial). This third condition (the “breath-hold” condition) was used in an attempt to non-invasively increase the strength of cardiac interoceptive signals^63^. An inspiratory breath-hold was specifically chosen since it leads to an increase in the overall volume within the chest cavity, bringing the heart against the chest wall, and potentially allowing one to more easily perceive palpitations at the point of maximum impulse (the physical location where the heart twists, moves forward and strikes against the anterior chest wall during systolic contraction of the heart muscle^27^. In healthy comparisons, we expected that performance on this series of tasks would be linked to the strength of the cardiac signal evoked during each condition, being lowest in the guessing condition, higher in the no-guessing condition where subjects only pressed when they confidently felt their heartbeat, and even higher in the breath-hold condition. To assess task compliance, we also included an exteroceptive control task. The parameters of this task were identical to the first heartbeat tapping condition, with the exception that participants were instructed to tap each time they heard an auditory tone. An average of 80 tones were presented to each subject to give a reasonable approximation to a typical heart rate (reflecting the mid-point of the normal heart rate range of 60–100 beats per minute). To further mimic the physiological presentation of this exteroceptive signal, the number of tones presented to subjects was randomly jittered by ± 10% and presented in a pattern following a sin curve with a frequency of 13 cycles/minute (mimicking the range of respiratory sinus arrhythmia during a normal breathing range of 13 breaths per minute). This “tone tapping task” was completed between the first and second heartbeat tapping conditions.

In the initial version of the task, participant responses were recorded using a VMeter (http://www.vmeter.net/), which is a touch sensitive device that provides no tactile feedback, similar to a laptop touch pad. This was motivated by concerns that feedback from pressing a button might distract participants from their interoceptive sensations. During an initial pilot phase with 31 participants, we found that some participant responses were missed due to improper finger placement or pressing very lightly. At this point, we switched to using a standard Apple keyboard button in order to provide enough feedback that participants could tell when their responses were recorded. Twenty-one participants who used the VMeter (with data manually checked for quality) were included in the results we report here; 10 healthy comparisons, 10 with depression/anxiety, and 1 with substance use disorder. We also re-analyzed data without including these individuals, and confirmed that the pattern of results described below remained unchanged.

### Psychophysiological measurement

A lead-II electrocardiogram (EKG) was used to continuously assess the electrical timing of participants' heartbeats throughout the task. Three EKG leads were connected to a MP150 unit (Biopac Systems, Inc., Santa Barbara). In addition, pulse plethysmography (PPG) was used to assess the physical timing of the pulse wave emanating from the systolic contraction of the heart, via a pulse oximeter attached to the earlobe. Response times were collected using a task implemented in PsychoPy, with data collection synchronized via a parallel port interface.

### Analysis

EKG and behavioral response data were scored using in-house developed MATLAB code. Each participant’s pulse transit time (PPT) was estimated as the median delay between the R wave and the corresponding positive inflection in the earlobe PPG signal. Potentially perceivable heartbeats were defined as occurring at the onset of systole as defined by each participant’s pulse transit time (PTT), with 200 ms used as a reasonable estimate for any participants without usable PTT data (according to previous estimates for the ear PTT^64^).

### Perceptual measure

Perceptual experience was assessed by calculating a measure of the variability in the time between an individual's taps (indexing subjective experience) and each heartbeat or tone signal (indexing objective events), which we term "beat-to-tap consistency". This perceptual measure was motivated by the fact that, to be reliable in tapping with the same delay relative to the occurrence of a heartbeat (under conditions in which the heartbeats themselves have variable timing), one would need to be accurately sensing the heartbeat signal. In contrast, the more variable the delay from beat-to-beat, the more random one’s perception (and subsequent tapping behavior) should be in relation to the occurrence of each beat.

**Figure 1:**
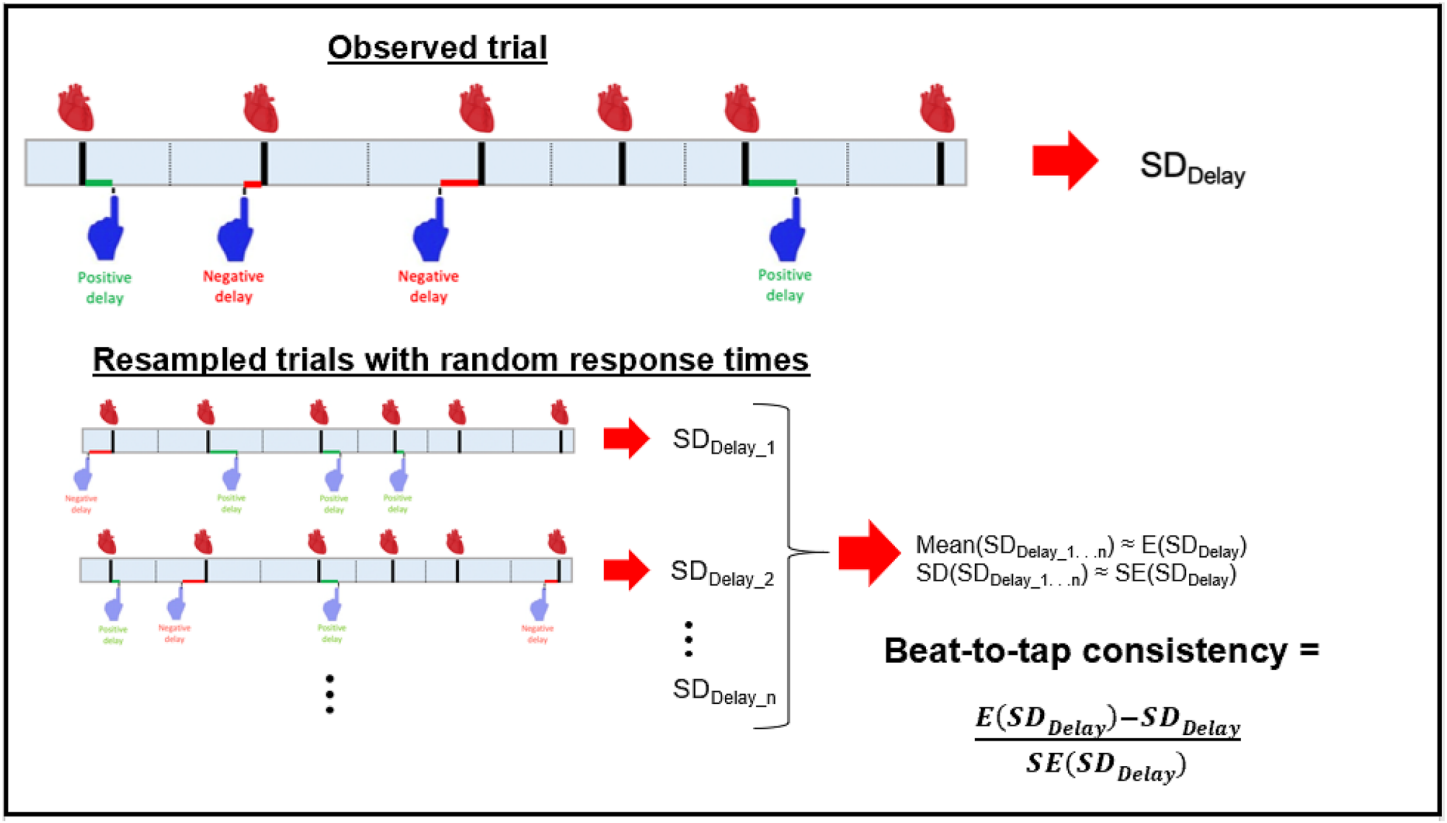
Illustration of how beat-to-tap consistency was calculated as a measure of reliability in the temporal relationship between participants’ heartbeats (shown as thick black lines with heart images above them) and taps (shown as blue hands, with either positive or negative temporal distances from each heartbeat) that was uncorrelated with their heart rate. This involved first estimating the distribution of expected (standard deviation; SD) values under random tapping using a participant’s actual recorded heartbeats and actual number of responses, placed randomly (indicated below by partially transparent hand images) using a uniform distribution for the trial over large numbers of simulated trials (here n = 1000 trials). Then a participant’s actual tapping behavior was converted into a Z-score by subtracting it from the mean and dividing by the standard deviation of the estimated distribution (see main text for more details).

The beat-to-tap consistency measure was calculated by first assigning each response to the nearest heartbeat (or tone) and calculating the time difference between the two—such that each response was assigned a response time, which could be positive or negative. The decision to use the nearest heartbeat (or tone) event, rather than the previous event as used by Ludwick-Rosenthal et al.^16^, was based on the observation that some participants tended to respond preemptively to repeated salient events (for an illustration, see Supplementary Fig. S1). First, we calculated the standard deviation of the response times. This measure, however, was expected to be (and was; see Supplementary Fig. S2) correlated with actual heart rate, due to the fact that faster heart rates yield shorter windows during which a participant may respond, so that a participant tapping randomly will tend to appear more precise. We estimated the distribution of expected values under random tapping using a participant’s actual recorded heart beats and actual number of responses, placed randomly using a uniform distribution for the minute trial. We then converted a participant’s actual tapping behavior into a Z-score by subtracting the mean and dividing by the standard deviation of the estimated distribution (see Fig. 1). In this way, we calculated a perceptual measure that was corrected for, and no longer correlated with (see Supplementary Fig. S2), actual heart rate: the beat-to-tap consistency. Note that, as a result, the beat-to-tap consistency measure also does not depend on individual differences in reaction times (e.g., as would be a concern if one instead simply measured the average temporal distance between heartbeats/tones and taps).

### Statistical analysis

Linear mixed effects models were run in R accounting for potential group differences in age, sex, their interaction with group, medication status, and tapping consistency within the tone trial (“tone-to-tap consistency”; TTC): y ~ Age × Group + Sex × Group + TTC + Medication status + Trial × Group + (1 + trial|participant), where y = beat-to-tap consistency values for the heartbeat tapping conditions. This allowed us to assess whether perceptual responses changed as a function of the trial condition and whether that change differed across groups (i.e., a Group by Trial interaction). TTC was included to account for potential individual differences in reaction times upon detecting a sensory signal generally (or other possible individual response idiosyncrasies unrelated to the interoception-specific aspects of task performance). Trial was coded as a continuous variable with values of 1 for the guessing trial, 2 for the no-guessing trial, and 3 for the no-guessing + breath hold trial. This decision was motivated by the idea that perceptual ability should improve during trials where subjects confidently feel their heartbeat (no-guessing condition) and should further increase with homeostatic perturbation during the breath-hold. Post-hoc tests then allowed us to interpret those effects to test the possibility that perceptual responses would progressively increase across the 3 task conditions in healthy participants but not in the patient groups. Our initial analysis of beat-to-tap consistency compared healthy participants to patients transdiagnostically. We then re-ran this analysis separating the depression/anxiety and substance use groups to assess the possibility of differences associated with distinct primary diagnoses. After confirming these results, we then ran analogous models to assess change across conditions in: number of taps and heartbeats, self-reported confidence in task performance, self-reported heartbeat intensity, self-reported difficulty performing the task, and counting accuracy. Heartbeat counting accuracy, a measure commonly used in prior cardioception research, was also determined using a modified version of the standard formula from the heartbeat counting task^17^—in this case, replacing the number counted with the number of taps. These were performed to assess different potential explanations for our main findings with beat-to-tap consistency.

### Exploratory machine learning analyses

In addition to testing for group differences in the analysis described above, we also chose to investigate individual differences in the slope of change in beat-to-tap consistency across all diagnostic groups. To do so, we ran a simpler model: y ~ Trial + (1 + trial | participant) that allowed us to extract a coefficient (random slope) for each individual characterizing the degree of change in beat-to-tap consistency across conditions (interoceptive modulation ability; IMA). We then used this individual difference measure in an exploratory machine learning analysis to assess its relationship to task-specific measures as well as a large number of other clinical measures (150 predictors in total) gathered from the Tulsa 1000 data set. These measures are listed in Supplementary Information, and they were chosen due to their potential relevance to symptoms and risk factors for psychiatric conditions.

The ensemble machine learning approach we used was identical to that used in Ref.^65^, and the reader is referred there (and to Supplementary Information) for additional details. Briefly, our approach combined predictions from multiple standard machine learning methods: elastic net (ENET), support vector regression (SVR), and random forest (RF). It then subsequently combined the predictions across methods by stacking or meta ensemble^66–68^. Performance was assessed using a nested cross-validation procedure.

In addition to model performance, we also assessed variable importance (VI) using stacking. Here, each individual machine learning method provided a measure of importance for each variable. Different individual methods had different importance measures: absolute values of regression coefficients were used for ENET, “out-of-bag” mean square error obtained by permutation was used for RF; and a “filter” approach (https://github.com/topepo/caret/blob/master/pkg/caret/R/filterVarImp.R) was used for SVR. Finally, single VI values for each variable—scoring how much that variable could account for IMA values—were computed by scaling each individual importance score (between 0 and 100) and then taking an average of the importance values of each method, weighted by the performance of each method (see Supplementary Information for details). Since univariate analyses are more commonly reported in the literature than the proposed machine learning approach, we also computed Pearson correlation coefficients, 95% confidence intervals, and FDR corrected p-values for comparison purposes.

## Results

Complete information on sample size, demographics, and symptom screening measure scores is provided in Table 1. Separate ANOVAs showed significant differences between groups in age (*F* (2, 407) = 4.76, *p* = 0.009), but no difference in body mass index (BMI; *F* (2, 393) = 1.2, *p* = 0.301). A chi-squared analysis also showed significant differences in sex composition between groups (χ^2^ (2) = 16.29, *p* < 0.001). Age and sex were thus controlled for in our main analyses below.

**Table 1:** Mean and standard deviation for clinical and demographic variables.

Clinical and demographic variables	Healthy comparisons	Depression/anxiety	Substance use	*p*-value*
N =	53	221	136	
Age	31.99 (10.97)	36.23 (11.17)	33.70 (8.87)	0.009
Sex (male)	28 (53%)	62 (28%)	60 (44%)	< 0.001
PHQ-9	0.89 (1.30)	12.57 (4.98)	6.70 (5.82)	< 0.001
OASIS	1.32 (1.88)	9.69 (3.46)	5.84 (4.78)	< 0.001
DAST-10	0.09 (0.30)	0.66 (1.41)	7.49 (2.19)	< 0.001
Body mass index (BMI)	27.64 (5.52)	28.79 (5.49)	28.23 (4.59)	0.301
Median Pulse Transit Time (s)	0.20 (0.02)	0.19 (0.02)	0.20 (0.02)	0.106

*p-values correspond to the results of ANOVAs comparing the three groups.

There was some loss of participants between conditions due primarily to there being too few responses to estimate beat-to-tap consistency during missing trials. Table 2 reports the final number of participants included for beat-to-tap consistency analyses in each condition, the descriptive statistics for beat-to-tap consistency by group and condition, as well as descriptive statistics by condition for the other task-related variables.

**Table 2:** Mean and standard deviation for task-related variables.

Panel A	Healthy comparisons	Depression/anxiety	Substance use	*p*-value*
**Guessing condition**				
N =	53	221	136	
Number of taps	68.15 (29.20)	65.25 (31.24)	65.96 (36.73)	0.847
Number of heartbeats	66.47 (9.13)	70.24 (10.31)	70.42 (10.07)	**0.036**
Average delay between heartbeat and tap	− 0.00 (0.05)	0.00 (0.04)	0.01 (0.06)	0.301
Self-reported difficulty^a^	57.53 (22.86)	53.73 (25.92)	47.96 (25.31)	0.032
Self-reported confidence	32.49 (19.20)	39.03 (21.11)	43.49 (23.27)	**0.006**
Self-reported intensity	20.51 (16.73)	28.75 (22.71)	36.68 (24.25)	**< 0.001**
Counting accuracy	0.70 (0.33)	0.66 (0.29)	0.63 (0.43)	0.4
Beat-to-tap consistency	0.01 (1.11)	0.44 (1.06)	0.52 (1.47)	**0.034**

Panel B	Healthy comparisons	Depression/anxiety	Substance use	*p*-value
**No-guessing condition**				
N =	41	182	122	
Number of taps	24.41 (22.87)	29.27 (23.64)	35.30 (25.13)	**0.02**
Number of heartbeats	64.85 (10.07)	69.41 (10.21)	70.27 (10.00)	**0.012**
Average delay between heartbeat and tap	0.00 (0.15)	-0.01 (0.10)	0.00 (0.07)	0.732
Self-reported difficulty	65.93 (27.48)	63.64 (29.86)	54.42 (29.43)	**0.014**
Self-reported confidence	33.76 (24.64)	39.62 (27.17)	40.71 (25.92)	0.338
Self-reported intensity	24.02 (19.31)	30.59 (25.77)	36.20 (26.26)	**0.019**
Counting accuracy	0.36 (0.28)	0.40 (0.30)	0.47 (0.31)	**0.045**
Beat-to-tap consistency	0.56 (2.23)	0.38 (1.68)	0.48 (1.75)	0.79

Panel C	Healthy comparisons	Depression/anxiety	Substance use	*p*-value
**Breath hold condition**				
N =	36	169	113	
Number of taps	32.72 (21.73)	31.45 (24.64)	38.51 (24.79)	**0.056**
Number of heartbeats	68.72 (9.74)	70.67 (10.35)	73.26 (11.03)	**0.038**
Average delay between heartbeat and tap	0.01 (0.12)	0.01 (0.11)	0.01 (0.08)	0.929
Self-reported difficulty	49.17 (26.49)	54.37 (25.04)	42.16 (27.55)	**0.001**
Self-reported confidence	47.47 (25.57)	50.40 (24.93)	52.09 (24.76)	0.614
Self-reported intensity	42.58 (27.33)	47.18 (27.55)	50.94 (27.29)	0.242
Counting accuracy	0.47 (0.28)	0.41 (0.27)	0.49 (0.28)	**0.037**
Beat-to-tap consistency	1.58 (2.46)	0.66 (1.66)	0.68 (1.17)	**0.007**

Panel D	Healthy comparisons	Depression/anxiety	Substance use	*p*-value
**Tone condition**				
N =	53	220	135	
Number of taps	77.68 (1.52)	77.54 (4.22)	79.24 (25.10)	0.557
Number of heartbeats	65.45 (10.66)	71.06 (10.34)	70.45 (10.15)	**0.002**
Average delay between tone and tap	0.01 (0.09)	0.00 (0.07)	-0.01 (0.06)	0.298
Self-reported difficulty	18.57 (16.15)	23.65 (21.21)	27.19 (23.13)	**0.039**
Self-reported confidence	76.91 (14.45)	75.66 (18.45)	74.33 (18.91)	0.643
Self-reported intensity	85.25 (13.89)	83.64 (16.70)	79.53 (18.18)	**0.038**
Counting accuracy	0.99 (0.02)	0.99 (0.05)	0.94 (0.32)	0.068
Beat-to-tap consistency	9.02 (3.51)	8.63 (3.48)	7.72 (4.20)	**0.037**

*p-values correspond to the results of ANOVAs comparing the three groups.

^a^Higher values indicate greater difficulty.

**Figure 2:**
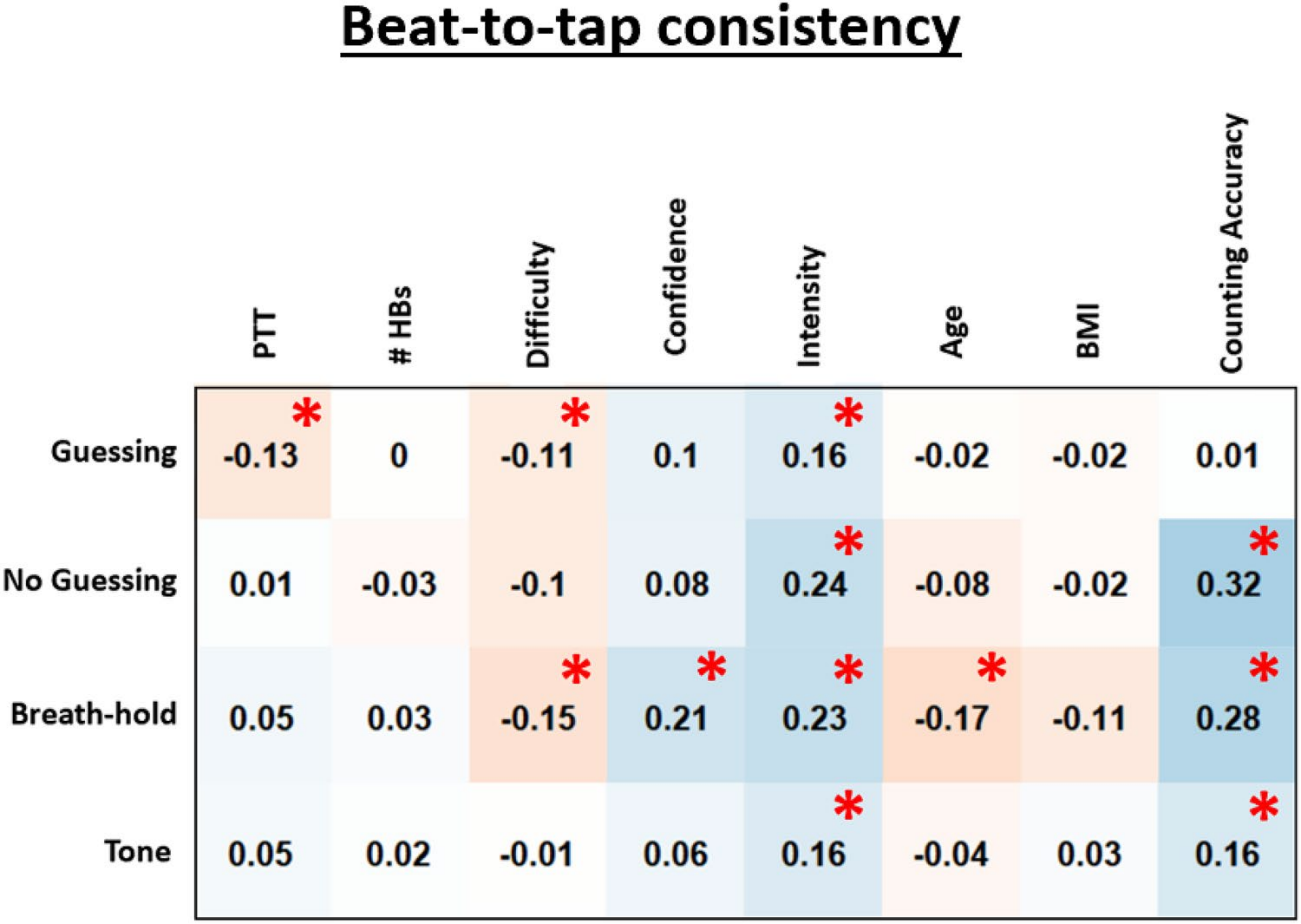
Exploratory Pearson correlations between beat-to-tap consistency by condition (rows), interoceptive accuracy via the standard heartbeat counting formula (counting accuracy), and other self-report and task-relevant variables across task conditions. *PTT *median pulse transit time, *#HBs *number of heartbeats during the task condition, *BMI *body mass index. For reference, significant correlations at p < 0.05 (uncorrected) are marked with red asterisks.

Pearson correlations between beat-to-tap consistency, counting accuracy, and other task-relevant variables are shown in Fig. 2. As can be seen there, beat-to-tap consistency showed significant (but fairly weak) correlations with counting accuracy, self-reported confidence, intensity, and difficulty. For an analogous figure showing these relationships for counting accuracy, see Supplementary Fig. S3, which showed a similar pattern of results.

### Breath-hold manipulation check

To check whether the breath-hold had the intended effect, we used all available data (including those for which beat-to-tap consistency could not be calculated) and compared the no-guessing and breath-hold conditions. As expected, relative to the no-guessing conditions, the breath-hold condition was associated with greater: number of taps (*t*(811) = 2.01, *p* = 0.04), heart rate (*t*(805) = 2.27, *p* = 0.02), self-reported confidence in task performance (*t*(802) = 5.06, *p* < 0.001)), self-reported heartbeat intensity (*t*(797) = 7.98, *p* < 0.001), as well as less self-reported task difficulty (*t*(801) = 5.01, *p* < 0.001)), across groups.

### Linear mixed effects analyses

**Figure 3:**
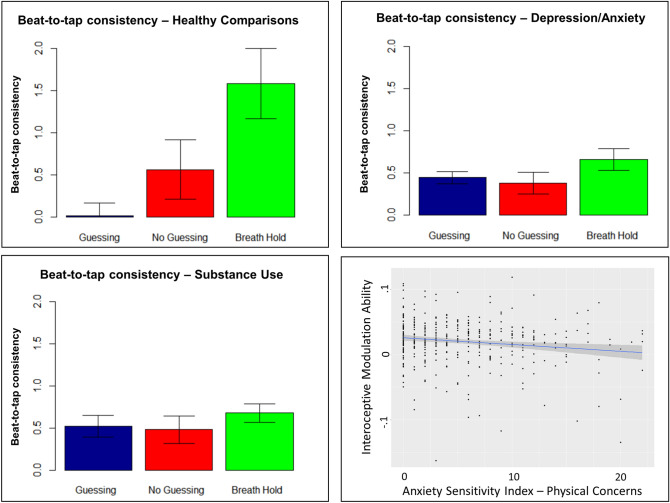
Bar plots showing the mean and standard error of beat-to-tap consistency by condition and group. There was an interaction between task condition and group, reflecting a pattern in which beat-to-tap consistency increased from the guessing and no-guessing conditions to the breath hold condition within healthy comparisons, whereas this measure remained unchanged within the two clinical groups. The bottom right plot shows that higher anxiety sensitivity scores for physical concerns (such as uncomfortable bodily sensations) were associated with lower interoceptive modulation ability (i.e., individual slope values reflecting how beat-to-tap consistency changed from the guessing to no-guessing to breath-hold conditions).

In our linear mixed effects (LME) analysis of beat-to-tap consistency with the transdiagnostic grouping, we observed a significant effect of sex (*F* (1, 394) = 11.16 *p* < 0.001; greater beat-to-tap consistency in males), medication status (*F* (1, 388) = 8.72, *p* = 0.003; lower beat-to-tap consistency in medicated individuals), tone-to-tap consistency (*F* (1, 376) = 4.76, *p* = 0.03; positive relationship), trial condition (*F* (1, 379) = 19.29, *p* < 0.001), group (*F* (1, 474) = 6.80, *p* = 0.009), and a significant interaction between trial condition and group (*F* (1, 379) = 11.18, *p* < 0.001). The effects of group and trial were accounted for by their interaction, which was driven by greater beat-to-tap consistency in the breath-hold condition in healthy comparisons than in patients (*t*(51) = 2.03, *p* = 0.048), and greater beat-to-tap consistency in the breath-hold condition than in the guessing (*t*(45) = 3.80, *p* < 0.001) condition in healthy comparisons but not in patients (see Fig. 3 for an illustration of the mean values by group and condition). A reversed pattern was also seen in the guessing condition, where lower beat-to-tap consistency was observed in the healthy comparisons than in the patient group (*t*(73) = 2.76, *p* = 0.007). No differences were seen in the no-guessing condition. Given the unexpected effect of medication status, in Supplementary Information we explored whether this effect might be primarily due to either antidepressant or anxiolytic medications. In the guessing and breath-hold conditions, reduced beat-to-tap consistency was only seen in those taking both antidepressants and anxiolytics in combination, while both medications separately and in combination were associated with lower beat-to-tap consistency in the no-guessing condition.

Results were similar when re-performing this analysis separating patients into the two diagnostic groups, with each group showing the same behavior pattern. Each of the previously found effects were still present: sex (*F* (1, 386) = 11.6352, *p* < 0.001; greater beat-to-tap consistency in males), medication status (*F* (1, 388) = 8.50, *p* = 0.004; lower beat-to-tap consistency in medicated individuals), tone-to-tap consistency (*F* (1, 373) = 4.51, *p* = 0.03; positive relationship), trial condition (*F* (1, 378) = 16.62, *p* < 0.001), group (*F* (2, 449) = 3.47, *p* = 0.03), and a group by trial interaction (*F* (2, 378) = 5.62, *p* = 0.004). Here the interaction was due to (1) greater beat-to-tap consistency in the breath-hold condition in healthy comparisons than in both depression/anxiety (*t*(57) = 2.0, *p* = 0.056; marginal) and substance use (*t*(55) = 2.01, *p* = 0.049), and (2) no significant differences in beat-to-tap consistency between trial conditions in either patient group (while beat-to-tap consistency was greater in the breath-hold condition than the guessing condition in healthy controls; as reported in the transdiagnostic analysis above; see Fig. 3). A reversed pattern was again seen in the guessing condition, where beat-to-tap consistency was lower in healthy comparisons than in depression/anxiety (*t*(77) = 2.5, *p* = 0.01) and substance use (*t*(126) = 2.5, *p* = 0.01). No differences were seen in the no-guessing condition.

Full results for the analogous LMEs—including number of taps, number of heartbeats, self-reported difficulty, confidence, and intensity, and counting accuracy—are also detailed in Supplementary Information. These analyses revealed significant trial type by group interactions with respect to number of heartbeats, difficulty, and counting accuracy. Further inspection suggested that the heart rate result was driven by a pattern in which heart rate increased in the breath-hold condition relative to the guessing and no-guessing conditions in healthy participants and substance users, whereas the depression/anxiety group did not show these differences, and instead showed greater heart rate in the guessing than no-guessing conditions (heart rate was also higher in the patient groups than in healthy participants in the guessing and no guessing conditions). The counting accuracy result was driven by a pattern in which counting accuracy was higher in the guessing condition than the other two conditions for all groups, but that substance users had higher counting accuracy than the other two groups in the no-guessing condition, and also had higher counting accuracy than the depression/anxiety group in the breath-hold condition. The difficulty result was driven by a pattern in which healthy participants reported less difficulty in the breath-hold condition than the guessing condition, whereas no differences in difficulty were present between these conditions in either patient group (i.e., mirroring the beat-to-tap consistency results). Healthy participants also reported less difficulty in the breath-hold condition than the depression/anxiety group, while they reported more difficulty in the guessing and no-guessing conditions than substance users (see Supplementary Information for full results).

Our subsequent exploratory machine learning analysis revealed that a model with all demographic, clinical, neuropsychological, and task variables only explained 1% of the variance in IMA slope values (i.e. which measures how much beat-to-tap consistency increased from the guessing to no guessing to breath-hold condition for each participant). The top 10 variables with highest VI from highest to lowest (and associated univariate correlation values) are shown in Table 3.

**Table 3:** Machine learning results.

Variable importance	Variable	r
1	Physical concerns subscale of the anxiety sensitivity index	− 0.17
2	PROMIS^69^ satisfaction subscale	0.12
3	Subjectively rated heartbeat intensity in the no-guessing condition	0.11
4	The state subscale of the state trait anxiety inventory	0.01
5	Age	− 0.13
6	CDDR positive reinforcement	0.08
7	PROMIS informational support subscale	0.13
8	PHQ-9 scale	− 0.13
9	PROMIS physical functioning scale	0.13
10	PROMIS ABI social scale	0.12

Top 10 variables of importance and associated univariate correlations in accounting for variance in interoceptive modulation ability.

## Discussion

This study probed cardiac interoceptive awareness across a large sample of patients with various psychiatric conditions spanning the depression, anxiety, and substance use disorder categories. The employed paradigms assessed whether different experimental conditions were associated with different levels of interoceptive awareness, with a predicted increase in afferent signal strength during a breath-hold perturbation relative to resting conditions. This breath-hold perturbation appeared effective, as it decreased self-reported difficulty and increased self-reported confidence, perceived heartbeat intensity, observed heart rate, and number of taps. Healthy volunteers showed the expected increase in beat-to-tap consistency in the breath-hold condition relative to the two resting (guessing and no-guessing) conditions, whereas the patient groups exhibited a blunted response. Consistent with this, only healthy volunteers reported significant reductions in difficulty from the guessing to breath-hold conditions. No group difference was seen in the no-guessing condition. And, unexpectedly, lower beat-to-tap consistency in the guessing condition was observed in healthy individuals than in the clinical groups. Notably, individuals on psychiatric medication showed lower beat-to-tap consistency, but group differences remained significant when accounting for this effect. Taken together, these data support the notion that psychiatric patients have a less flexible dynamic range of interoceptive processing that manifests most prominently during states of physiological perturbation.

The current study builds on previous bodies of work that have separately shown associations between mood, anxiety, and substance use disorders and interoceptive processing deficits (for reviews see Refs.^47,70–73^). For example, one recent study found that a transdiagnostic patient sample (including affective disorders, schizophrenia, and personality disorders) showed lower subjective interoceptive confidence on both a heartbeat tracking task and a heartbeat discrimination task, as well as lower objective performance on the latter^74^. Other previous cardiac perception studies have reported that depressed individuals exhibit reduced accuracy on a heartbeat counting task^75–77^, and that performance is negatively correlated with depressive symptoms^78^ as well as associated with both lower positivity and poorer decision-making^75^. As our assessment approach does not suffer from some of the limitations of heartbeat counting tasks discussed above, these results may provide a stronger confirmation that interoceptive processing abnormalities are present in these clinical populations, especially under the context of physiological perturbation. Notably, the relationship between beat-to-tap consistency and heartbeat counting accuracy was weak, and the same pattern of changes in counting accuracy between task conditions was not found, suggesting that these are largely separate measures. However, an exploratory machine learning analysis of relationships between several putatively relevant clinical variables explained very little of the variability in beat-to-tap consistency across the different conditions.

The current results provide evidence of reduced flexibility in interoceptive processing in individuals with depression, anxiety, and substance use disorders relative to healthy comparisons, such that—while healthy individuals demonstrated changes in beat-to-tap consistency across conditions—the different task contexts had no effect on this interoceptive awareness measure in the patient groups. Based on these results, it will be worth considering in future work what mechanisms could account for such deficits in the flexibility with which interoceptive signals are processed. For example, perhaps inflexible interoceptive processing arises in part as a result of central modulation deficits in some patients (e.g., a relative inability to internally adjust the way afferent interoceptive signals are attended to under changes in afferent signal strength). Alternatively, as interoceptive signals are thought to play a role in directing cognitive processes toward homeostatically relevant stimuli^79^, perceptual insensitivity to the modulation of interoceptive signals might also contribute to reductions in the adaptive allocation of cognitive resources to the task. It is worth noting however, that because performance did not improve in the breath-hold condition in the patient populations, this could further suggest a kind of perceptual processing rigidity independent of cognitive modulation, where interoceptive percepts are relatively insensitive to (or less constrained by) changes in bottom-up signals from the body^80^.

One recently influential theoretical framework through which these findings might be viewed is the predictive processing framework^81–83^. This framework views the brain as a probabilistic inference machine capable of evaluating the most likely external causes of noisy sensory data, and of selecting both skeletomotor and visceromotor (and cognitive) actions based on predicted reductions in uncertainty, predicted metabolic demands, and predicted returns to internal and external conditions consistent with preferences and continued survival. A central concept within this framework is the notion of precision, which refers to implicit beliefs about the reliability of different internally and externally generated signals. One type of precision—sensory precision—corresponds to the estimated reliability with which patterns of sensory input provide evidence for one causal event outside of the brain over others. In any perceptual domain, the brain may learn "default" sensory precision estimates reflecting the baseline reliability of particular sensory signals, encoded in the pattern of synaptic strengths within sensory cortices. These precision estimates can be updated when afferent signal properties change, and they can also be adjusted through top-down (e.g., goal-directed) modulatory influences over these synaptic strengths emanating from higher cortical structures.

In the context of the present study, our temporally derived measure of heartbeat perception (i.e., heartbeat tapping) during the guessing condition could be understood as reflecting the default interoceptive sensory precision estimates acquired (or inherited) by a given individual with little influence from condition-specific cognitive modulation. Low values in this condition would therefore suggest that afferent signals originating from objective heartbeats are not encoded as reliable signals by an individual's brain. Notably, our beat-to-tap consistency results in the breath-hold condition suggest that objectively increasing signal reliability did lead to updated (increased) precision estimates in healthy participants. But, it did not lead to this type of online updating in the depressed/anxious or substance use groups. In contrast to these lower-level afferent processing mechanisms, increases in accuracy due to the no-guessing instruction could instead reflect the efficacy with which task instruction-specific cognitive modulation could alter the pattern of synaptic strengths within interoceptive processing cortices such that afferent signals were treated as more reliable. The fact that patient groups had higher interoceptive consistency in the guessing condition, no difference in the no-guessing condition, and lower consistency in the breath-hold condition suggests a type of fixed, unadjustable estimate of cardiac signal reliability—whereas healthy individuals could adjust this estimate in a flexible, dynamic way.

Assuming that afferent cardiac signals are in fact sufficiently reliable, increases in the precision-weighting of such signals would be expected to increase accuracy in heartbeat perception. Alternatively, the no-guessing instruction could have produced goal-directed changes in the thresholding of interoceptive evidence, such that a stronger signal was necessary to drive the decision to tap the button. This latter mechanism is more consistent with changes in behavior leading to a reduced number of false positives without a reduced number of false negatives. While the specific underlying mechanism remains unclear, our results seem to indicate a generally reduced ability to modulate interoceptive signal processing in those with depression/anxiety and substance use disorders. Testing these different mechanistic hypotheses further would likely require a combination of explicit computational modeling, measurement of the associated neural circuitry (e.g. via functional neuroimaging and/or electroencephalography), and to perhaps include pharmacological or other physiological manipulations aimed at altering relevant neural modulatory systems (for recent work using this type of computational modelling approach, see Refs.^59,84^).

Consistent with the central modulation mechanism hypothesized above, it is noteworthy that individuals on psychotropic medications (which act on central neuromodulators) showed lower beat-to-tap consistency. It is unclear whether this is a counterproductive side effect of psychotropic medications or whether it could be beneficial (e.g., reducing awareness of unpleasant bodily sensations), and we don’t interpret it further here. However, there are a few other previous findings which could relate to this result. For example, one study found that a serotonergic receptor antagonist reduced activation in regions of the insula associated with interoception^85^, which could pertain to reduced perceptual accuracy. Another study found higher heart rate and lower heart rate variability in individuals on antipsychotic medications, and higher self-reported bodily awareness in individuals on antidepressant medications^74^. Yet another study showed that acute administration of a serotonin reuptake inhibitor increased metacognitive awareness of performance accuracy on a cardiac interoception task^86^. However, as the aforementioned findings have mainly related to self-report or otherwise cognitive measures distinct from perceptual accuracy itself (as in this study), future work will be necessary to confirm the effects we observed and how they relate to these previous findings.

The exploratory machine learning analyses did not reveal strong predictive relationships between our individual subject measure of interoceptive processing modulation ability (IMA; i.e., the slope of change in beat-to-consistency across conditions for each subject) and several demographic, clinical, and neuropsychological variables—only accounting for approximately 1% of the variance in IMA scores. Thus, while we observed significant group differences between healthy and clinical samples, further individual variation was not well explained by differences in symptom severity or other clinically relevant continuous measures we examined. In other words, despite the group effect, high between-subject variability may have prevented further (cross-validated, out-of-sample) predictive power at the individual level. It may be noteworthy, however, that the top variable of importance was the physical concerns subscale of the anxiety sensitivity index^87^. This subscale measures the degree to which perceived bodily sensations trigger states of fear and anxiety, and our results showed that having lower IMA was associated with greater anxiety sensitivity in this domain. One possibility is that lower IMA corresponds to a reduced ability to adjust the influence of afferent bodily signals when such signals become more or less noisy. If so, benign fluctuations in bodily sensations could be more easily misinterpreted as meaningful signals of danger and therefore promote greater anxiety. However, the replicability of this finding, and its correct interpretation, will need to be confirmed in future research. The same is true with respect to the relationships observed with other variables of high importance in our results. The associations with depression (PHQ-9 scores), age, and perceived heartbeat intensity appear consistent with what one might expect based on previous studies^75–78^; whereas those with several other variables are less clear. The univariate associations between IMA and these other variables were lower than for anxiety sensitivity, however; given that the model as a whole performed poorly, these results do not warrant strong interpretation, and suggest that that other (perhaps genetic/environmental) factors could play a greater role.

There are several limitations to the present study. First, there was substantial data loss when arriving at the final analysis set (17–32%), primarily because some individuals did not feel their heart beat (and thus didn’t tap) a sufficient number of times to calculate beat-to-tap consistency. We would expect that this limitation could be overcome by utilizing a more robust physiological perturbation (e.g., inspiratory breathing loads, adrenergic modulation of the heartbeat with isoproterenol), but for our purposes such methodologies were not available for inclusion in the Tulsa 1000 project. Second, our sample had considerable clinical heterogeneity. While this improves representativeness of clinical populations in the community, it does limit our ability to interpret results with respect to specific diagnostic categories. Third, while the breath-hold perturbation appears to have been effective, it is notably weaker than the stronger, more invasive perturbations used in previous studies (e.g., isoproterenol infusion or carbon dioxide inhalation challenges). Finally, the Tulsa 1000 study from which our sample was taken did not include measures of emotion regulation ability. Given the relationship between emotion regulation and visceral regulation (e.g. engaging cognitive/behavioral strategies to reduce emotional arousal), an important future question may be to test whether reduced interoceptive modulation ability could relate to more difficulties in emotion regulation.

## Conclusions

Our results provide evidence that individuals with depression/anxiety and substance use disorders show a perceptual insensitivity to the modulation of interoceptive signals, highlighting unique individual differences in the manner in which interoceptive information is processed by humans in different cognitive and physiological contexts. Future studies should further investigate the clinical relevance of these differences in flexibility of interoceptive modulation.

## Supplementary Information

Supplementary Information.

## References

[1] Khalsa SS (2018). Interoception and mental health: A roadmap. Biol. Psychiatry Cogn. Neurosci. Neuroimaging.

[2] Barlow D, Allen L, Choate M (2016). Toward a unified treatment for emotional disorders—Republished article. Behav. Ther..

[3] Boswell JF (2019). A preliminary naturalistic clinical case series study of the feasibility and impact of interoceptive exposure for eating disorders. Behav. Res. Ther..

[4] Craske MG (2011). A cognitive-behavioral treatment for irritable bowel syndrome using interoceptive exposure to visceral sensations. Behav. Res. Ther..

[5] Price CJ (2019). Immediate effects of interoceptive awareness training through Mindful Awareness in Body-oriented Therapy (MABT) for women in substance use disorder treatment. Subst. Abus..

[6] Zucker NL (2019). Feeling and body investigators (FBI): ARFID division-An acceptance-based interoceptive exposure treatment for children with ARFID. Int. J. Eat. Disord..

[7] Goossen B, van der Starre J, van der Heiden C (2019). A review of neuroimaging studies in generalized anxiety disorder: "So where do we stand?". J. Neural Transm. (Vienna).

[8] Etkin A, Wager TD (2007). Functional neuroimaging of anxiety: A meta-analysis of emotional processing in PTSD, social anxiety disorder, and specific phobia. Am. J. Psychiatry.

[9] Goodkind M (2015). Identification of a common neurobiological substrate for mental illness. JAMA Psychiatry.

[10] Smith R, Thayer JF, Khalsa SS, Lane RD (2017). The hierarchical basis of neurovisceral integration. Neurosci. Biobehav. Rev..

[11] Berntson GG, Khalsa SS (2020). Neural circuits of interoception. Trends Neurosci..

[12] Garfinkel S, Seth A, Barrett A, Suzuki K, Critchley H (2014). Knowing your own heart: Distinguishing interoceptive accuracy from interoceptive awareness. Biol. Psychol..

[13] Simmons W (2013). Keeping the body in mind: Insula functional organization and functional connectivity integrate interoceptive, exteroceptive, and emotional awareness. Hum. Brain Mapp..

[14] Brener J, Kluvitse C (1988). Heartbeat detection: Judgments of the simultaneity of external stimuli and heartbeats. Psychophysiology.

[15] Katkin ES, Morell MA, Goldband S, Bernstein GL, Wise JA (1982). Individual differences in heartbeat discrimination. Psychophysiology.

[16] Ludwick-Rosenthal R, Neufeld RW (1985). Heart beat interoception: A study of individual differences. Int. J. Psychophysiol..

[17] Schandry R (1981). Heart beat perception and emotional experience. Psychophysiology.

[18] Phillips GC, Jones GE, Rieger EJ, Snell JB (1999). Effects of the presentation of false heart-rate feedback on the performance of two common heartbeat-detection tasks. Psychophysiology.

[19] Ring C, Brener J, Knapp K, Mailloux J (2015). Effects of heartbeat feedback on beliefs about heart rate and heartbeat counting: A cautionary tale about interoceptive awareness. Biol. Psychol..

[20] Windmann S, Schonecke OW, Frohlig G, Maldener G (1999). Dissociating beliefs about heart rates and actual heart rates in patients with cardiac pacemakers. Psychophysiology.

[21] Zamariola G, Maurage P, Luminet O, Corneille O (2018). Interoceptive accuracy scores from the heartbeat counting task are problematic: Evidence from simple bivariate correlations. Biol. Psychol..

[22] Murphy J (2018). Knowledge of resting heart rate mediates the relationship between intelligence and the heartbeat counting task. Biol. Psychol..

[23] Ainley V, Tsakiris M, Pollatos O, Schulz A, Herbert BM (2018). Interoceptive Accuracy Scores are Problematic: Evidence from Simple Bivariate Correlations"-The empirical data base, the conceptual reasoning and the analysis behind this statement are misconceived and do not support the authors' conclusions. Biol. Psychol..

[24] Corneille O, Desmedt O, Zamariola G, Luminet O, Maurage P (2020). A heartfelt response to Zimprich et al. (2020), and Ainley et al. (2020)'s commentaries: Acknowledging issues with the HCT would benefit interoception research. Biol. Psychol..

[25] Khalsa SS, Lapidus RC (2016). Can interoception improve the pragmatic search for biomarkers in psychiatry?. Front. Psychiatry.

[26] Barsky AJ (1998). Palpitations and cardiac awareness after heart transplantation. Psychosom. Med..

[27] Khalsa SS, Rudrauf D, Sandesara C, Olshansky B, Tranel D (2009). Bolus isoproterenol infusions provide a reliable method for assessing interoceptive awareness. Int. J. Psychophysiol..

[28] Avery JA (2014). Major depressive disorder is associated with abnormal interoceptive activity and functional connectivity in the insula. Biol. Psychiatry.

[29] Berner LA (2018). Altered interoceptive activation before, during, and after aversive breathing load in women remitted from anorexia nervosa. Psychol. Med..

[30] Berner LA (2019). Altered anticipation and processing of aversive interoceptive experience among women remitted from bulimia nervosa. Neuropsychopharmacology.

[31] DeVille DC (2018). The neural bases of interoceptive encoding and recall in healthy adults and adults with depression. Biol. Psychiatry Cogn. Neurosci. Neuroimaging.

[32] Kerr KL, Moseman SE, Avery JA, Bodurka J, Simmons WK (2017). Influence of visceral interoceptive experience on the brain's response to food images in anorexia nervosa. Psychosom. Med..

[33] Stewart JL (2019). Interoceptive attention in opioid and stimulant use disorder. Addict. Biol..

[34] Strigo IA (2013). Altered insula activation during pain anticipation in individuals recovered from anorexia nervosa: Evidence of interoceptive dysregulation. Int. J. Eat. Disord..

[35] Domschke K, Stevens S, Pfleiderer B, Gerlach AL (2010). Interoceptive sensitivity in anxiety and anxiety disorders: An overview and integration of neurobiological findings. Clin. Psychol. Rev..

[36] Ehlers A, Breuer P (1996). How good are patients with panic disorder at perceiving their heartbeats?. Biol. Psychol..

[37] Willem Van der Does AJ, Antony MM, Ehlers A, Barsky AJ (2000). Heartbeat perception in panic disorder: A reanalysis. Behav. Res. Ther..

[38] Charney DS, Heninger GR, Jatlow PI (1985). Increased anxiogenic effects of caffeine in panic disorders. Arch. Gen. Psychiatry.

[39] Dillon DJ, Gorman JM, Liebowitz MR, Fyer AJ, Klein DF (1987). Measurement of lactate-induced panic and anxiety. Psychiatry Res..

[40] Gurguis GN, Vitton BJ, Uhde TW (1997). Behavioral, sympathetic and adrenocortical responses to yohimbine in panic disorder patients and normal controls. Psychiatry Res..

[41] Pohl R (1988). Isoproterenol-induced panic attacks. Biol. Psychiatry.

[42] Rassovsky Y, Kushner MG (2003). Carbon dioxide in the study of panic disorder: Issues of definition, methodology, and outcome. J. Anxiety Disord..

[43] Schunck T (2006). Functional magnetic resonance imaging characterization of CCK-4-induced panic attack and subsequent anticipatory anxiety. Neuroimage.

[44] Asmundson GJ, Stein MB (1994). Triggering the false suffocation alarm in panic disorder patients by using a voluntary breath-holding procedure. Am. J. Psychiatry.

[45] Eggart M, Lange A, Binser MJ, Queri S, Muller-Oerlinghausen B (2019). Major depressive disorder is associated with impaired interoceptive accuracy: A systematic review. Brain Sci..

[46] Jakubczyk A (2019). Interoceptive accuracy and interoceptive sensibility in individuals with alcohol use disorder-Different phenomena with different clinical correlations?. Drug Alcohol Depend..

[47] Paulus MP, Stewart JL (2014). Interoception and drug addiction. Neuropharmacology.

[48] Stewart JL (2014). You are the danger: Attenuated insula response in methamphetamine users during aversive interoceptive decision-making. Drug Alcohol Depend..

[49] Berk L (2015). Under pressure: Adolescent substance users show exaggerated neural processing of aversive interoceptive stimuli. Addiction.

[50] Canales-Johnson A (2015). Auditory feedback differentially modulates behavioral and neural markers of objective and subjective performance when tapping to your heartbeat. Cereb. Cortex.

[51] Victor TA (2018). Tulsa 1000: A naturalistic study protocol for multilevel assessment and outcome prediction in a large psychiatric sample. BMJ Open.

[52] Insel T (2010). Research domain criteria (RDoC): Toward a new classification framework for research on mental disorders. Am. J. Psychiatry.

[53] Kroenke K, Spitzer RL, Williams JB (2001). The PHQ-9: Validity of a brief depression severity measure. J. Gen. Intern. Med..

[54] Norman SB, Cissell SH, Means-Christensen AJ, Stein MB (2006). Development and validation of an Overall Anxiety Severity And Impairment Scale (OASIS). Depress. Anxiety.

[55] Staley D, el-Guebaly, N.  (1990). Psychometric properties of the Drug Abuse Screening Test in a psychiatric patient population. Addict. Behav..

[56] Morgan JF, Reid F, Lacey JH (2000). The SCOFF questionnaire: A new screening tool for eating disorders. West J. Med..

[57] Smith, R. *et al.* Imprecise action selection in substance use disorder: Evidence for active learning impairments when solving the explore-exploit dilemma. *Drug Alcohol Depend.* (2020) **(in press)**. 10.1016/j.drugalcdep.2020.108208 PMC7502502 32801113

[58] Smith, R. *et al.* Greater decision uncertainty characterizes a transdiagnostic patient sample during approach-avoidance conflict: A computational modeling approach. *J. Psychiatry Neurosci.* (2020) **(in press)**. 10.1503/jpn.200032 PMC7955838 33119490

[59] Smith R, Kuplicki R, Feinstein J, Forthman KL, Stewart JL, Paulus MP (2020). A Bayesian computational model reveals a failure to adapt interoceptive precision estimates across depression, anxiety, eating, and substance use disorders. PLoS. Comput. Biol..

[60] Sheehan DV (1998). The Mini-International Neuropsychiatric Interview (M.I.N.I.): The development and validation of a structured diagnostic psychiatric interview for DSM-IV and ICD-10. J. Clin. Psychiatry.

[61] Pollatos O, Herbert BM, Matthias E, Schandry R (2007). Heart rate response after emotional picture presentation is modulated by interoceptive awareness. Int. J. Psychophysiol..

[62] Desmedt O, Luminet O, Corneille O (2018). The heartbeat counting task largely involves non-interoceptive processes: Evidence from both the original and an adapted counting task. Biol. Psychol..

[63] Fitz-Clarke JR (2007). Computer simulation of human breath-hold diving: Cardiovascular adjustments. Eur. J. Appl. Physiol..

[64] Allen J, Murray A (2003). Age-related changes in the characteristics of the photoplethysmographic pulse shape at various body sites. Physiol. Meas..

[65] Ekhtiari H, Kuplicki R, Yeh HW, Paulus MP (2019). Physical characteristics not psychological state or trait characteristics predict motion during resting state fMRI. Sci. Rep..

[66] Wolpert DH (1992). Stacked generalization. Neural Netw..

[67] Breiman L (1996). Stacked regressions. Mach. Learn..

[68] Van der Laan, M. J., Polley, E. C. & Hubbard, A. E. Super learner. *Stat. Appl. Genet. Mol. Biol.* **6**. 10.2202/1544-6115.1309 17910531

[69] Flynn KE (2013). Development of the NIH PROMIS^®^ Sexual Function and Satisfaction measures in patients with cancer. J. Sex. Med..

[70] Barrett LF, Quigley KS, Hamilton P (2016). An active inference theory of allostasis and interoception in depression. Philos. Trans. R. Soc. Lond. B Biol. Sci..

[71] Paulus MP, Stein MB (2010). Interoception in anxiety and depression. Brain Struct. Funct..

[72] Paulus MP, Tapert SF, Schulteis G (2009). The role of interoception and alliesthesia in addiction. Pharmacol. Biochem. Behav..

[73] Verdejo-Garcia A, Clark L, Dunn BD (2012). The role of interoception in addiction: A critical review. Neurosci. Biobehav. Rev..

[74] Critchley, H. D. *et al.* Transdiagnostic expression of interoceptive abnormalities in psychiatric conditions. *medRxiv*. doi:10.1101/19012393 (2019).

[75] Furman D, Waugh C, Bhattacharjee K, Thompson R, Gotlib I (2013). Interoceptive awareness, positive affect, and decision making in major depressive disorder. J. Affect. Disord..

[76] Terhaar J, Viola FC, Bär K-J, Debener S (2012). Heartbeat evoked potentials mirror altered body perception in depressed patients. Clin. Neurophysiol..

[77] Dunn BD (2010). Can you feel the beat? Interoceptive awareness is an interactive function of anxiety- and depression-specific symptom dimensions. Behav. Res. Ther..

[78] Pollatos O, Traut-Mattausch E, Schandry R (2009). Differential effects of anxiety and depression on interoceptive accuracy. Depress. Anxiety.

[79] Barrett L, Satpute A (2013). Large-scale brain networks in affective and social neuroscience: Towards an integrative functional architecture of the brain. Curr. Opin. Neurobiol..

[80] Paulus MP, Feinstein JS, Khalsa SS (2019). An active inference approach to interoceptive psychopathology. Annu. Rev. Clin. Psychol..

[81] Clark, A. Surfing uncertainty: Prediction, action, and the embodied mind. (2015).

[82] Parr T, Friston K (2018). The anatomy of inference: Generative models and brain structure. Front. Comput. Neurosci..

[83] Teufel C, Fletcher PC (2020). Forms of prediction in the nervous system. Nat. Rev. Neurosci..

[84] Smith, R., Kuplicki, R., Teed, A., Upshaw, V., & Khalsa, S. S. Confirmatory Evidence that Healthy Individuals Can Adaptively Adjust Prior Expectations and Interoceptive Precision Estimates. In *Active Inference. IWAI 2020. Communications in Computer and Information Science* (eds. Verbelen, T., Lanillos, P., Buckley, C. L., & De Boom, C.) vol. 1326. 10.1007/978-3-030-64919-7_16. Preprint published on *bioRxiv*. 10.1101/2020.08.31.275594 (Springer, Cham, 2020).

[85] Stern ER (2019). High-dose ondansetron reduces activation of interoceptive and sensorimotor brain regions. Neuropsychopharmacology.

[86] Livermore, J. J. A. *et al.* Serotonergic effects on interoception. *bioRxiv*. doi:10.1101/2020.08.28.262550 (2020).

[87] Taylor S (2007). Robust dimensions of anxiety sensitivity: Development and initial validation of the Anxiety Sensitivity Index-3. Psychol. Assess..

