# Supplementary material for: Perceptual insensitivity to the modulation of interoceptive signals in depression, anxiety, and substance use disorders
Source: Sci Rep. 2021 Jan 22;11:2108. doi: 10.1038/s41598-021-81307-3 (PMC7822872; doi:10.1038/s41598-021-81307-3)
Supplement: Supplementary file 1 — Supplementary Information. [file 41598_2021_81307_MOESM1_ESM.pdf]

Supplementary Materials for:

Perceptual insensitivity to the modulation of interoceptive signals in depression, anxiety, and substance use disorders

Ryan Smith[1], Justin S. Feinstein[1,2], Rayus Kuplicki[1], Katherine L. Forthman[1], Jennifer L. Stewart[1,2], Martin P. Paulus[1,2], Tulsa 1000 Investigators[†], Sahib S. Khalsa[1,2]

[†]The **Tulsa 1000 Investigators** consortium includes the following additional contributors: Robin L. Aupperle, Ph.D.[1,2], Jerzy Bodurka, Ph.D.[1,2], Jonathan B. Savitz, Ph.D.[1,2], and Teresa A. Victor, Ph.D.[1].

[1]Laureate Institute for Brain Research, Tulsa, OK, USA

[2]Oxley College of Health Sciences, University of Tulsa, Tulsa, OK, USA

Clinical group details

The focus of this study was transdiagnostic. However, for secondary analysis of patient sub-groups, patients were divided based on primary diagnoses of 1) Major Depression and/or co-morbid anxiety disorders, or 2) substance use disorders. Individuals were not included in the substance use group if showing only alcohol or nicotine dependence (as was done in the Tulsa 1000 study from which our sample was drawn), or for whom substance use was not the primary diagnosis. Secondary diagnoses for these subgroups present in more than 5% of patients, as well as additional patient information, are detailed in Table S1. Information about medication use is shown in Table S2.

Table S1*. Secondary diagnoses present in more than 5% of patients within either subgroup.*

Diagnoses	Depression/Anxiety	Substance Use Disorders
**Current Major Depression**	24%	5%
**Lifetime Major Depression**	89%	51%
**Single Episode vs. Recurrent Major Depression**	Single Episode: 22%<br>Recurrent: 69%	Single Episode: 18%<br>Recurrent: 18%
**Social Phobia**	18%	13%
**Post-traumatic Stress Disorder**	19%	16%

**Panic Disorder**	19%	12%
**Generalized Anxiety Disorder**	39%	15%
**Cannabis Use**	~	29%
**Opioid Use**	~	27%
**Stimulant Use**	~	15%
**Alcohol Use**	10%	29%
**Opioid Use**	~	27%
**Sedative/Hypnotic Use**	~	8%

Table S2. *Percentage of major psychiatric medication classes taken by more than 5% of patients in either subgroup.*

Prescribed Medications	Depression/Anxiety	Substance Use Disorders
**Antidepressants**	54%	31%
**Anxiolytics**	34%	24%
**Antipsychotics**	~	9%
**Stimulants**	6%	~

Machine learning details

Because different machine learning methods make different assumptions, they can sometimes predict outcomes with different levels of accuracy; no single method is always superior to other methods with respect to predictive accuracy [1,2]. We addressed this problem using the "wisdom of crowds" approach [3] by combining predictions from multiple methods: elastic net (ENET), support vector regression (SVR), and random forest (RF), and then subsequently combined the predictions across methods by stacking or meta ensemble [4-6]. To assess the performance of stack ensembles in independent, unseen datasets, we also conducted nested cross-validation (nested CV) where the inner loop was used to build base and stacked models, and the outer loop was used to evaluate model performance. To increase the stability of the results, the entire nested CV procedure was repeated 20 times, allowing us to estimate confidence intervals. Additionally, since some methods require complete data, any missing values in the 150 predictors were imputed using k-nearest neighbors before entering the repeated nested CV.

In addition to model performance, we also assessed variable importance (VI) using stacking where each machine learning method provided a measure of importance for each variable. Different individual methods had different importance measures: absolute values of regression coefficients for ENET, "out-of-bag" mean square error obtained by permutation for RF; for SVR, a "filter" approach (https://github.com/topepo/caret/blob/master/pkg/caret/R/filterVarImp.R) was used. In this approach the response variable was regressed on each feature one a time by a loess (LOcally WEighted Scatter-plot Smoother) and the R-square was computed as the variable importance. These VI measures were scaled to between 0 and 100 for each individual model. Next, the stacked importance was computed as the weighted average of the importance across models using the weights determined by the stacking

model described above. This produced a single set of VI values for each stacked model in the outer loop of nested CV. VI was averaged across folds to obtain a single set of values. 20 random partitions were used (i.e. 20 repeats of nested CV), and 95% confidence intervals for VI were taken as each variable's mean importance ± 1.96 times its standard deviation.

Machine learning predictors

Table S3. *Full variable list for machine learning analyses (see supplemental material in [7] for detailed descriptions of each variable).*

**Demographic Variables**
Age
Sex
Education
Income
Race (White, Black, Hispanic, Native American, Other)
Medicated
**Task-Related Variables**
Number of heartbeats (for each task condition)
Self-reported difficulty, confidence, and intensity (for each task condition)
Body Mass Index (BMI)
Median Pulse Transit Time
Finger tapping test (average number of taps per 10 seconds with dominant hand)
**Self Report Scales**
World Health Organization Health and Work Performance Questionnaire (contains 8 subscale scores)
Multidimensional Assessment of Interoceptive Awareness (contains 8 subscale scores)
Anxiety Sensitivity Index (ASI; contains 3 subscale scores and 1 total score)
Patient Health Questionnaire (PHQ-9) score
Positive and Negative Affect Scale (PANAS) Attentiveness score
Positive and Negative Affect Scale (PANAS) Fatigue score
Positive and Negative Affect Scale (PANAS) Fear score
Positive and Negative Affect Scale (PANAS) Guilt score
Positive and Negative Affect Scale (PANAS) Hostility score
Positive and Negative Affect Scale (PANAS) Joviality score
Positive and Negative Affect Scale (PANAS) Negative Affect Total score
Positive and Negative Affect Scale (PANAS) Positive Affect Total score
Positive and Negative Affect Scale (PANAS) Sadness score
Positive and Negative Affect Scale (PANAS) Self Assurance score
Positive and Negative Affect Scale (PANAS) Serenity score

Positive and Negative Affect Scale (PANAS) Shyness score
Positive and Negative Affect Scale (PANAS) Surprise score
PROMIS Ability to Participate Social Activities score
PROMIS Anger score
PROMIS Anxiety score
PROMIS Depression score
PROMIS Positive Affect and Wellbeing score
PROMIS Nicotine Coping Expectancies score
PROMIS Nicotine Dependence score
PROMIS Nicotine Emotional Sensory Expectancies score
PROMIS Nicotine Health Expectancies score
PROMIS Nicotine Psychosocial Expectancies score
PROMIS Nicotine Social Motivations score
Ruminative Response Scale (RRS) score
State Trait Anxiety Inventory (STAI) State score
State Trait Anxiety Inventory (STAI) Trait score
Toronto Alexithymia Scale (TAS; 3 subscale scores and 1 total score)
Overall Anxiety Severity and Impairment Scale (OASIS)
PROMIS Emotional Support score
PROMIS Informational Support score
PROMIS Interest in Sexual Activities score
PROMIS Social Isolation score
PROMIS Social Satisfaction in Participation in Discretionary Social Activities score
PROMIS Social Satisfaction with Role score
Positive and Negative Affect Scale (PANAS) Fatigue score
PROMIS Applied Cognitive Abilities score
PROMIS Applied Cognitive General Concerns score
PROMIS Fatigue score
PROMIS Pain Behavior score
PROMIS Pain Interference score
PROMIS Physical Function score
PROMIS Sleep Disturbance score
PROMIS Sleep Related Impairment score
WHO Disability Assessment Screen (WHODAS) total score
Temporal Experience of Pleasure Scale (TEPS) Anticipatory score
Temporal Experience of Pleasure Scale (TEPS) Consummatory score
UPPS Impulsive Behavior Scale (UPPS-P) Lack of Perseveration score
UPPS Impulsive Behavior Scale (UPPS-P) Lack of Pre-meditation score
UPPS Impulsive Behavior Scale (UPPS-P) Negative Urgency score

UPPS Impulsive Behavior Scale (UPPS-P) Positive Urgency score
UPPS Impulsive Behavior Scale (UPPS-P) Sensation Seeking score
Customary Drinking and Drug Use Record (CDDR) Negative Reinforcement score
Customary Drinking and Drug Use Record (CDDR) Positive Reinforcement score
Drug Abuse Screening Test (DAST-10) score
PROMIS Alcohol Negative Consequences score
PROMIS Alcohol Negative Expectancies score
PROMIS Alcohol Positive Consequences score
PROMIS Alcohol Positive Expectancies score
PROMIS Alcohol Use score
Tulsa Head Injury Screen
**Full Variable Name (neuropsychological variables)**
Wide Range Achievement Test (WRAT) Reading
California Verbal Learning Test (CVLT) False Positives
California Verbal Learning Test (CVLT) Long Delay Cued Recall
California Verbal Learning Test (CVLT) Long Delay Free Recall
California Verbal Learning Test (CVLT) Long Delay Retention Vs Trial 5
California Verbal Learning Test (CVLT) Recognition
California Verbal Learning Test (CVLT) Semantic Clustering base
California Verbal Learning Test (CVLT) Short Delay Cued Recall
California Verbal Learning Test (CVLT) Short Delay Free Recall
California Verbal Learning Test (CVLT) Long Delay Retention Vs Trial 5
California Verbal Learning Test (CVLT) Total Intrusions
California Verbal Learning Test (CVLT) Total Repetitions
California Verbal Learning Test (CVLT) Trial 1 Scaled
California Verbal Learning Test (CVLT) Trial B Scaled
California Verbal Learning Test (CVLT) Trial 1 to 5 Scaled
Delis-Kaplan Executive Function System (DKEFS) Color-Word Inhibition color naming
Delis-Kaplan Executive Function System (DKEFS) Color-Word Inhibition color word reading scaled
Delis-Kaplan Executive Function System (DKEFS) Color-Word Inhibition reading comprehension scaled
Delis-Kaplan Executive Function System (DKEFS) Color-Word Inhibition errors inhibition switching scaled
Delis-Kaplan Executive Function System (DKEFS) Color-Word Inhibition errors cumulative rank
Delis-Kaplan Executive Function System (DKEFS) Color-Word Inhibition scaled
Delis-Kaplan Executive Function System (DKEFS) Color-Word Inhibition switching scaled
Delis-Kaplan Executive Function System (DKEFS) Color-Word Inhibition naming errors cumulative rank

Delis-Kaplan Executive Function System (DKEFS) Color-Word Inhibition Switching Vs Color Naming Scaled
Delis-Kaplan Executive Function System (DKEFS) Color-Word Inhibition Switching Vs Word Reading Scaled
Delis-Kaplan Executive Function System (DKEFS) Color-Word Inhibition Vs Color Naming Scaled
Delis-Kaplan Executive Function System (DKEFS) Color-Word Inhibition Vs Combined Naming Reading Scaled
Delis-Kaplan Executive Function System (DKEFS) Color-Word Inhibition Reading Errors Cumulative Rank
Wechsler Adult Intelligence Scale (WAIS-IV) backward digit span scaled
Wechsler Adult Intelligence Scale (WAIS-IV) forward digit span scaled
Wechsler Adult Intelligence Scale (WAIS-IV) letter number sequencing scaled
Wechsler Adult Intelligence Scale (WAIS-IV) total digit span scaled
Delis-Kaplan Executive Function System (DKEFS) verbal fluency category fluency
Delis-Kaplan Executive Function System (DKEFS) verbal fluency category switching
Delis-Kaplan Executive Function System (DKEFS) verbal fluency category switching total accuracy
Delis-Kaplan Executive Function System (DKEFS) verbal fluency letter fluency
Delis-Kaplan Executive Function System (DKEFS) verbal fluency repetition errors
Delis-Kaplan Executive Function System (DKEFS) verbal fluency repetition errors scaled
Delis-Kaplan Executive Function System (DKEFS) verbal fluency set loss errors
Delis-Kaplan Executive Function System (DKEFS) verbal fluency set loss errors scaled

Further linear mixed effects analyses

Here we report linear mixed effects analyses analogous to those reported in the main text (dividing patients into the two diagnostic sub-groups), but which focused on other task-relevant measures.

Number of heartbeats. We observed a significant effect of sex ($F(1, 394) = 5.92$, $p = .02$), a sex by group interaction ($F(2, 394) = 5.55$, $p = .004$), a significant effect of trial condition ($F(1, 399) = 17.13$, $p < .001$), and an interaction between group and trial condition ($F(2,399) = 4.95$, $p = .008$). Post-hoc t-tests showed the following pattern: 1) heart rate was greater in the breath-hold condition relative to the guessing and no-guessing conditions in healthy participants ($t(51) = 2.74$, $p = .008$; t(51) = 3.55, $p < .001$, respectively) and in substance users ($t(131) = 3.14$, $p = .002$; $t(131) = 3.70$, $p < .001$, respectively), whereas the depression/anxiety group did not show these differences, and instead showed greater heart rate in the guessing than no-guessing conditions ($t(219) = 2.41$, $p = .02$). 2) In both the guessing and no-guessing conditions, heart rate was lower in healthy participants than in both the depression/anxiety group ($t(87) = 2.63$, $p = .01$; $t(82) = 2.60$, $p = .01$, respectively) and the substance use group ($t(95) = 2.61$, $p = .01$; t(104) = 2.60, $p = .01$, respectively). Substance users also had marginally greater heart rate than healthy participants in the breath-hold condition ($t(103) = 1.90$, $p = .06$). Contrasts showed that the interaction

between sex and group reflected a pattern in which heart rate was higher in females than: 1) males in the substance use group (t-ratio(394) = 4.40, $p < .001$), and 2) males and females in the healthy and depression/anxiety group (t-ratios(394) between 2.73 and 3.2, ps between < .001 and .007).

Counting accuracy. We observed a significant effect of tone-to-tap consistency (F(1, 392) = 4.39, $p = .04$; positive relationship), trial condition (F(1,400) = 106.68, $p < .001$), and interactions between group and trial condition (F(2, 400) = 3.15, $p = .04$) and group and sex (F(2, 393) = 3.10, $p = .05$). Post-hoc t-tests showed that the group by trial interaction reflected the following: 1) counting accuracy was higher in the guessing condition than the other two conditions for all groups (ts between 3.93 and 11.4, $ps < .001$ for all), and higher in the breath-hold condition than in the no-guessing condition in healthy participants (t(51) = 2.9, $p = .006$). 2) Substance users had higher counting accuracy than healthy participants (t(105) = 3.18, $p = .002$) and the depression/anxiety group (t(273) = 2.96, $p = .003$) in the no-guessing condition, substance users had higher counting accuracy than the depression/anxiety group in the breath-hold condition (t(266) = 2.97, $p = .003$). Contrasts showed that the group by sex interaction reflected a pattern in which female substance users had greater counting accuracy than male substance users (t-ratio(395) = 2.96, $p = .04$), female healthy participants (t-ratio(391) = 2.44, $p = .02$), and depressed/anxious males and females (t-ratio(391) = 2.33, $p = .02$, t-ratio(393) = 3.38, $p < .001$, respectively).

Number of taps. We observed a significant effect of age (F(1, 391) = 9.55, $p = .002$; older age = fewer taps), medication status (F(1, 392) = 4.39, $p = .04$; more taps in medicated individuals), and trial condition (F(1, 401) = 266.08, $p < .001$; more taps in the guessing condition than the other two conditions, $p < .001$; and more taps in the breath-hold than no-guessing condition, $p = .02$), and a group by sex interaction (F(2,393) = 3.27, $p = .04$). Post-hoc contrasts showed that this reflected a greater number of taps in female substance users than male substance users (t-ratio(393) = 2.05, $p = .04$), healthy females (t-ratio(395) = 2.16, $p = .03$), and depressed/anxious females (t-ratio(396) = 3.72, $p < .001$).

Self-reported difficulty. We observed a significant effect of tone-to-tap consistency (F(1, 393) = 4.56, $p = .03$; positive relationship), trial condition (F(1, 400) = 4.86, $p = .03$; greater difficulty in the no-guessing condition than the other conditions, p < .001 each; and greater difficulty in the guessing than breath-hold condition, $p = .02$), and an interaction between group and trial condition (F(2, 400) = 3.53, $p = .03$). This reflected the following: 1) less difficulty in the breath-hold condition than the guessing condition in healthy participants (t(51) = 2.90, $p = .006$), but no differences between these conditions in either patient group. 2) Less difficulty in healthy participants than in the depression/anxiety group in the breath-hold condition (t(76) = 2.10, $p = .04$), more difficulty in healthy participants than in substance users in the guessing and no-guessing conditions (t(104) = 2.10, $p = .01$; t(76) = 2.10, $p = .04$, respectively), and more difficulty in the depression/anxiety group than in substance users across all conditions (ts between 2.10 and 3.10, ps between .002 and .04).

Self-reported confidence. We observed a significant effect of trial condition (F(1, 616) = 44.98, $p < .001$), reflecting greater confidence in the breath-hold condition than the guessing and no-guessing conditions (t(403) = 7.8, $p < .001$; t(402) = 7.10, $p < .001$, respectively).

Self-reported intensity. We observed a significant effect of trial condition (F(1,400) = 45.75, $p < .001$), reflecting greater intensity in the breath-hold condition than in the guessing and no-guessing conditions

($t(403) = 8.30$, $p < .001$; $t(402) = 11.20$, $p < .001$, respectively), and greater intensity in the guessing condition than in the no-guessing condition ($t(408) = 2.65$, $p = .008$).

Exploratory analyses of medication effects

As our main analyses showed that beat-to-tap consistency was lower in participants on medication, we explored here whether this effect might be selectively due to antidepressant or anxiolytic medications (i.e., the two major classes of medication taken in this sample; see Table S2). To do so, we conducted two sample t-tests comparing un-medicated individuals to individuals on antidepressants only, anxiolytics only, or on both medications. In the guessing condition, there were no significant effects of antidepressants or anxiolytics alone. In contrast, participants taking both medications together showed marginal evidence of lower beat-to-tap consistency than un-medicated individuals ($t(181) = 1.90$, $p = .06$). In the no-guessing condition, both antidepressants and anxiolytics separately, as well as in combination, were associated with marginally lower beat-to-tap consistency ($t(230) = 1.92$, $p = .055$; $t(55) = 1.93$, $p = .06$; $t(180) = 1.91$, $p = .06$, respectively). In the breath-hold condition, only those taking both anxiolytics and antidepressants in combination showed lower beat-to-tap consistency ($t(205) = 2.31$, $p = .02$).

Supplementary Figures

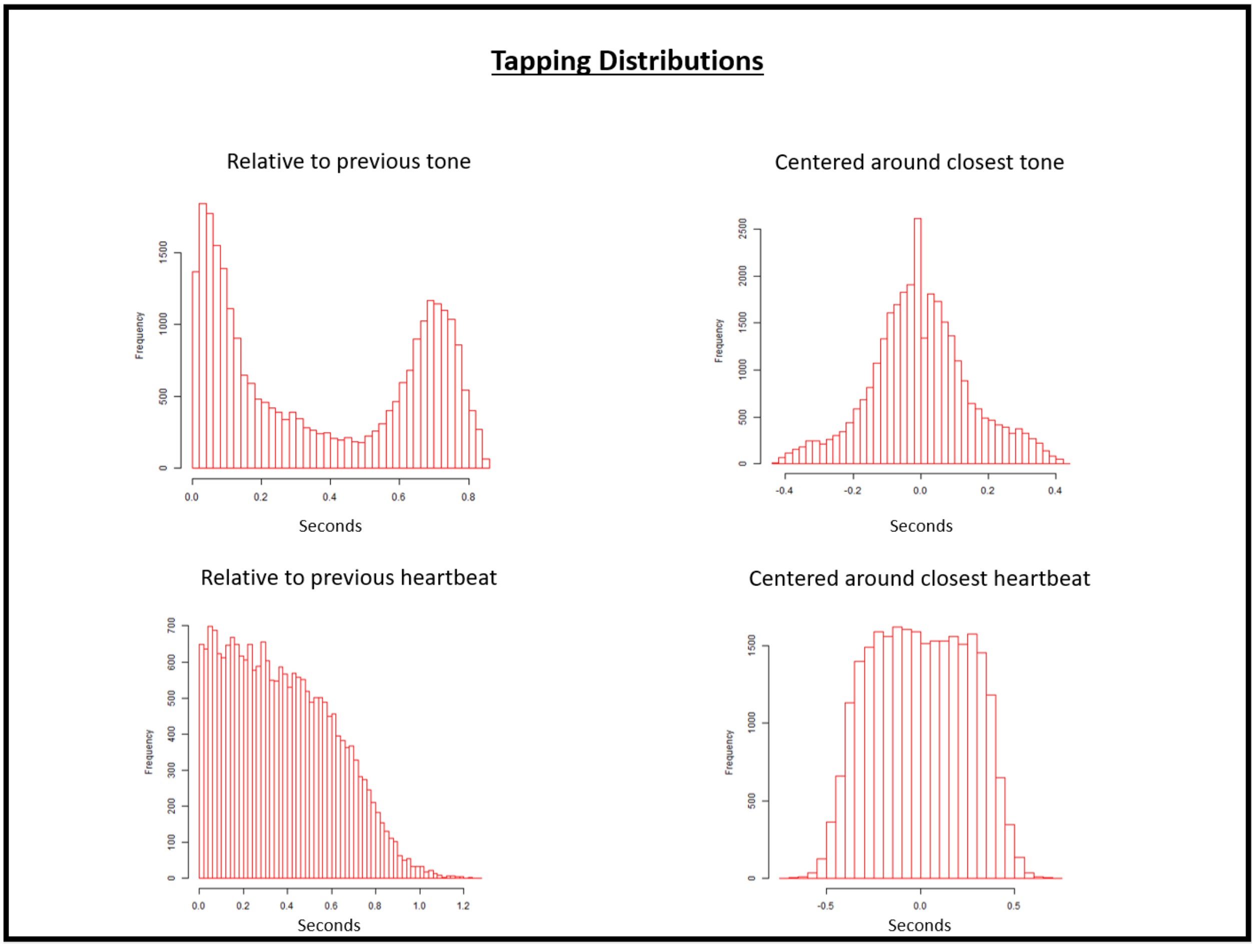

Figure S1. Illustration of the distribution of taps over time relative to either the previous tone/heartbeat or the closest tone/heartbeat (timepoint 0 = tone/heartbeat on the x-axes). As can be seen most clearly in the upper left plot, with the precise tone signal, taps tended to cluster into either anticipatory (just before the next tone) or reactive (just after the previous tone) temporal locations.

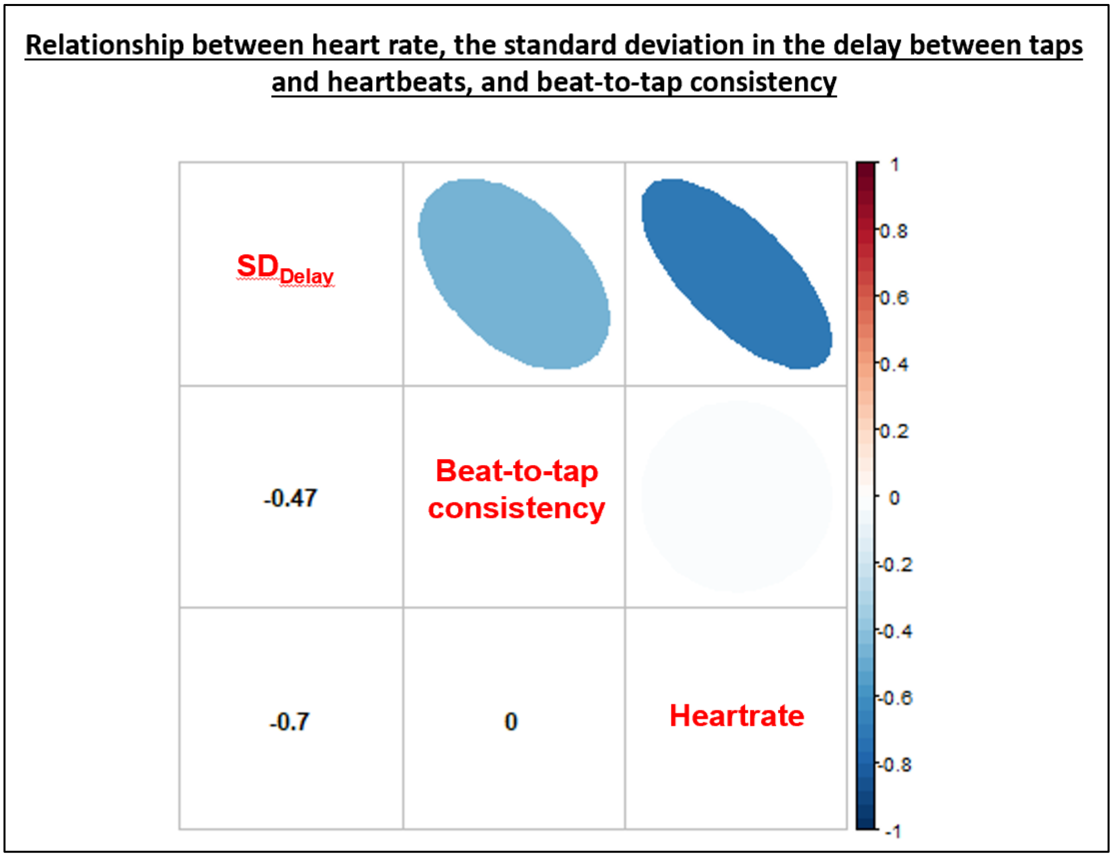

Figure S2. Illustration correlation between precision and heartrate, and how beat-to-tap consistency is unrelated to heartrate. Correlations are across all participants and conditions.

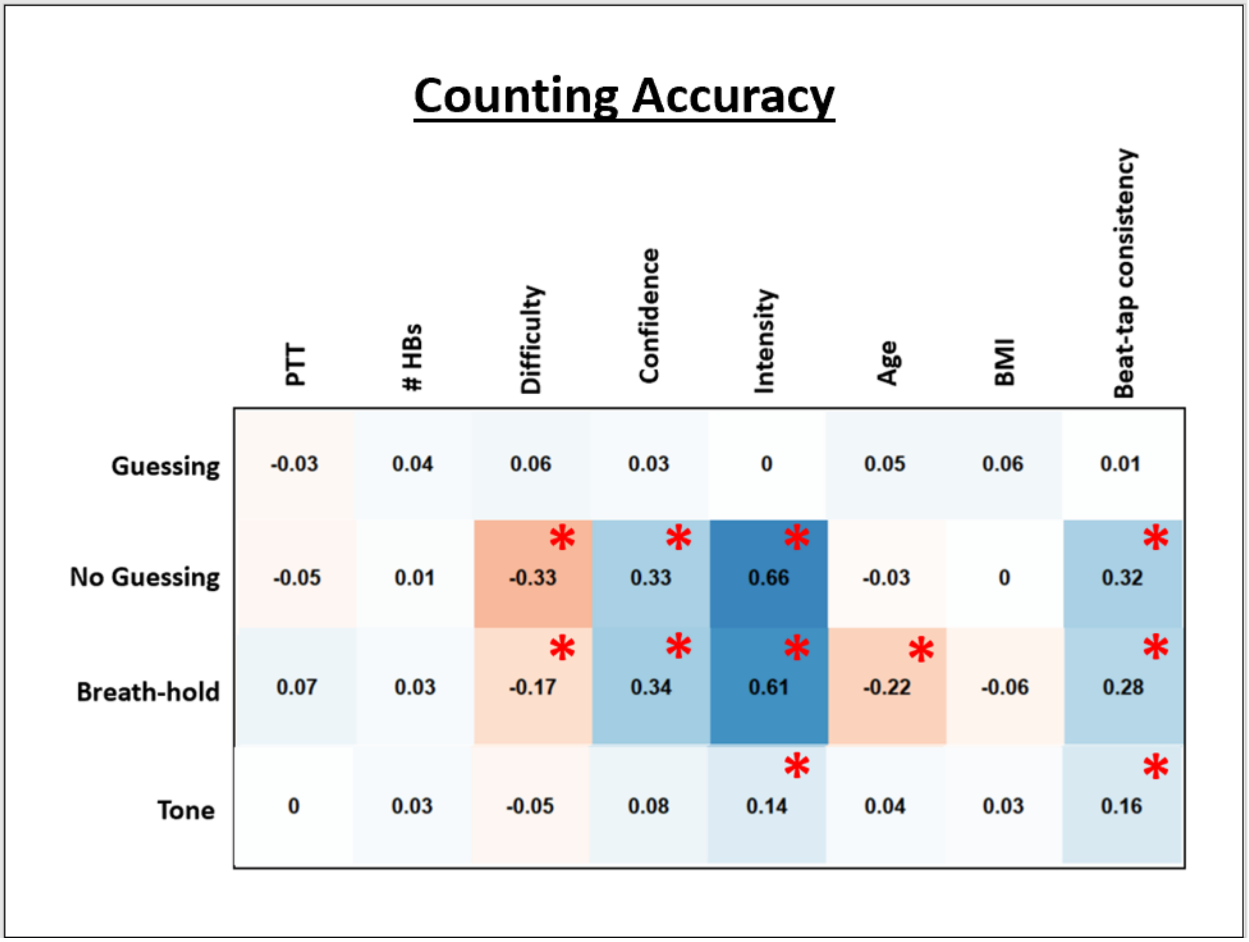

Figure S3. Pearson correlations between heartbeat counting accuracy (using the Schandry formula applied to number of reported taps), beat-to-tap consistency, and self-report and other task-relevant variables across task conditions. PTT = median pulse transit time, #HBs = number of heartbeats during the task condition, BMI = body mass index. For reference, correlations at $p < .05$ are marked with red asterisks.

References:

1 Benjamin, A. S. *et al.* Modern Machine Learning as a Benchmark for Fitting Neural Responses. *Front Comput Neurosci* **12**, 56, doi:10.3389/fncom.2018.00056 (2018).

2 Glaser, J. I., Benjamin, A. S., Farhoodi, R. & Kording, K. P. The roles of supervised machine learning in systems neuroscience. *Prog Neurobiol* **175**, 126-137, doi:10.1016/j.pneurobio.2019.01.008 (2019).

3 Marbach, D. *et al.* Wisdom of crowds for robust gene network inference. *Nat Methods* **9**, 796-804, doi:10.1038/nmeth.2016 (2012).

4 Wolpert, D. H. Stacked generalization. *Neural networks* **5**, 241-259 (1992).

5 Breiman, L. Stacked regressions. *Machine learning* **24**, 49-64 (1996).

6 Van der Laan, M. J., Polley, E. C. & Hubbard, A. E. Super learner. *Statistical applications in genetics and molecular biology* **6**.
7 Victor, T. A. *et al.* Tulsa 1000: a naturalistic study protocol for multilevel assessment and outcome prediction in a large psychiatric sample. *BMJ open* **8**, e016620 (2018).
